# The Length of Membership and Loneliness of Older Adults in Village Programs

**DOI:** 10.1093/geroni/igab046.140

**Published:** 2021-12-17

**Authors:** Qiuchang (Katy) Cao, Christine Happel, Katie White, Holly Dabelko-Schoeny

**Affiliations:** 1 The Ohio State University, Columbus, Ohio, United States; 2 Clintonville-Beechwold Community Resources Center, Clintonville-Beechwold Community Resources Center, Ohio, United States; 3 Ohio State University, Age-Friendly Columbus and Franklin County, Ohio, United States; 4 The Ohio State University, The Ohio State University, Ohio, United States

## Abstract

Villages are consumer-driven programs supporting older adults to age in their own homes while staying socially connected through service referrals, coordination, and the organization of social activities. Although previous studies demonstrated an increase of perceived social support among Village members over time, few studies tested how Village membership influence older adults’ loneliness. To address this gap, a total of 112 members from four Village programs in a Midwest Metropolitan area completed a cross-sectional pilot survey on their social well-being between January and March 2020. The age of participants ranged from 51 to 92 years old (M=72.30, SD=8.38), over 74% of participants were female and over 88% of participants identified as White/Caucasian. The relationship between the 20-item UCLA loneliness scale and length of Village membership was roughly linear according to the Loess Curve. The scores of the UCLA scale range from 20-80 and higher scores indicate higher loneliness. The Cronbach’s alpha of the UCLA loneliness scale was 0.86 in the sample, indicating good internal consistency. The average loneliness score of the sample was 38.45, resembling the average of community-living older adults. Regression results suggested that a one-year increase in village membership was associated with approximately two points reduction in loneliness, holding all else constant. Being female, a racial/ethnic minority, retired, a driver, and having higher frequencies of socializing with friends and neighbors were associated with lower levels of loneliness among Village members. This pilot study provides initial support for the social impact of Villages and informs future larger sample longitudinal studies.

